# Ablation of *Atp5if1* impairs metabolic reprogramming and proliferation of T lymphocytes and compromises mouse survival

**DOI:** 10.1016/j.isci.2024.109863

**Published:** 2024-05-03

**Authors:** Inés Romero-Carramiñana, Sonia Dominguez-Zorita, Pau B. Esparza-Moltó, José M. Cuezva

**Affiliations:** 1 Departamento de Biología Molecular, Centro de Biología Molecular Severo Ochoa, Consejo Superior de Investigaciones Científicas-Universidad Autónoma de Madrid (CSIC-UAM), 28049 Madrid, Spain; 2 Centro de Investigación Biomédica en Red de Enfermedades Raras (CIBERER) ISCIII, Madrid, Spain; 3 Instituto de Investigación Hospital 12 de Octubre, Universidad Autónoma de Madrid, Madrid, Spain

**Keywords:** Biological sciences, Physiology, Molecular biology, Immunology

## Abstract

T cells experience metabolic reprogramming to an enhanced glycolysis upon activation. Herein, we have investigated whether ATPase Inhibitory Factor 1 (IF1), the physiological inhibitor of mitochondrial ATP synthase, participates in rewiring T cells to a particular metabolic phenotype. We show that the activation of naive CD4^+^ T lymphocytes both *in vitro* and *in vivo* is accompanied by a sharp upregulation of IF1, which is expressed only in Th1 effector cells. T lymphocytes of conditional CD4^+^-IF1-knockout mice display impaired glucose uptake and flux through glycolysis, reducing the biogenesis of mitochondria and cellular proliferation after activation. Consequently, mice devoid of IF1 in T lymphocytes cannot mount an effective Th1 response against bacterial infection compromising their survival. Overall, we show that the inhibition of a fraction of ATP synthase by IF1 regulates metabolic reprogramming and functionality of T cells, highlighting the essential role of IF1 in adaptive immune responses.

## Highlights

• Activated CD4^+^ IF1-KO cells prevent ATP synthase inhibition impairing glucose uptake
• Activated CD4^+^ IF1-KO cells limit aerobic glycolysis and mitochondrial biogenesis
• The proliferation of activated CD4^+^ IF1-KO cells is impaired *in vitro* and *in vivo*
• CD4^+^ IF1-KO mice have an arrested Th1 immune response against bacterial infection

## Graphical abstract

**Figure:**
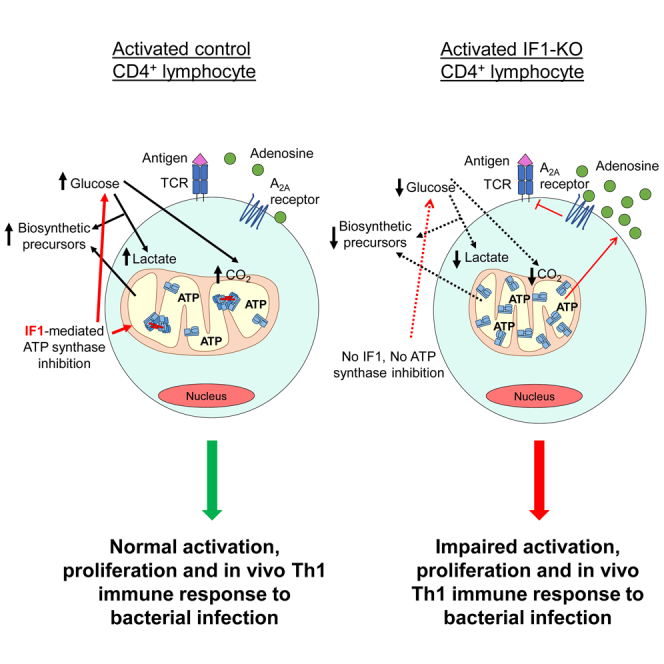

## Introduction

The ATP synthase is the rotatory engine of the inner mitochondrial membrane (IMM) that synthetizes cellular ATP using the proton electrochemical gradient generated by the respiratory chain in a process known as oxidative phosphorylation (OXPHOS).^1^^,^^2^ However, the ATP synthase can also operate in a reverse hydrolytic mode in conditions of mitochondrial de-energization, such as in ischemia, in order to maintain the mitochondrial membrane potential.^3^ The enzyme is composed of the F1-catalytic domain joined to the Fo-rotor domain and a peripheral stalk.^2^^,^^4^ In addition to other protein components of the IMM^5^^,^^6^^,^^7^ and the intrinsic membrane curvature provided by cardiolipin,^8^ the oligomerization^4^^,^^9^ and rotation^10^ of ATP synthase also contribute to bend the lipid bilayer at cristae rims to increase the surface area of the IMM for an efficient OXPHOS. Moreover, the ATP synthase also forms part of the permeability transition pore (PTP)^11^ and of the mitochondrial hub that controls cellular signaling^12^^,^^13^^,^^14^^,^^15^ involved in gearing cell death or adaptation to changing physiological cues. Conformational changes triggered by different anions and agents modulate the versatility of ATP synthase activities. In this regard, the ATPase Inhibitory Factor 1 (IF1) is the main physiological inhibitor of ATP synthase described so far.^16^^,^^17^^,^^18^^,^^19^

IF1 is a small protein encoded in the nuclear genome by the *ATP5IF1* gene which, after cleavage of the N-terminal mitochondrial targeting sequence, becomes the mature protein that associates into the active homodimeric form.^19^ The N-terminal region of mature IF1 is structurally disordered and, upon its binding into the α/β interface of the F1-catalytic domain of the enzyme, blocks its rotatory catalysis.^20^ *In vitro* experiments support that changes in matrix pH regulate the activity of IF1 as inhibitor of the enzyme. IF1 has been proposed to be active under acidic pH, which promotes its dimerization, while matrix alkalinization inactivates IF1 by favoring formation of tetramers.^2^^,^^21^^,^^22^^,^^23^ In addition, protein kinase A-mediated phosphorylation of S39 in IF1 also inactivates IF1 as an inhibitor of both the ATP synthase and hydrolase activities of ATP synthase because it prevents the interaction with the enzyme.^24^

Using a variety of cell lines and mouse models of loss and gain of function of IF1^17^^,^^25^^,^^26^^,^^27^^,^^28^^,^^29^^,^^30^^,^^31^ and *in vivo* pharmacologic approaches,^24^ it has been documented that the mitochondrial dosage of IF1 concertedly controls *in vivo* the overall catalytic activities of mitochondrial ATP synthase (synthesis and hydrolysis of ATP), supporting the idea that in some mouse tissues and in cells in culture there is a fraction of IF1-inhibited ATP synthase under normal phosphorylating conditions. The coexistence of active and inactive ATP synthase in cristae has been further supported after finding that different microdomains in the mitochondrion show heterogeneous membrane potential (ΔΨm), with microdomains of high ΔΨm resulting from the interaction of IF1 with ATP synthase.^28^

A fundamental aspect of the inhibition of ATP synthase by IF1 is that it promotes the metabolic reprogramming of the cells to an enhanced glycolysis by limiting the ATP provided by OXPHOS in the presence of oxygen.^17^^,^^25^^,^^26^^,^^29^^,^^32^ The activation of glycolysis is essential for the production of metabolic intermediates, reducing power and energy that support cellular proliferation.^33^^,^^34^^,^^35^ In this regard, it is worth noting that highly proliferative cancer cells^17^^,^^29^^,^^32^ show high rates of aerobic glycolysis and high expression levels of IF1 when compared to non-tumorigenic or differentiated cells. Likewise, stemness factor-mediated nuclear reprogramming of somatic cells induces the upregulation of IF1 concurrently with the activation of glycolysis,^36^^,^^37^ which is needed for the reprogramming of somatic cells to induced pluripotent stem cells (iPSC).^38^ On the contrary, silencing and degradation of IF1 is required for the differentiation of human mesenchymal stem cells into osteocytes.^39^ Overall, the expression of IF1 is advantageous for highly proliferative cells because it mediates, in the presence of oxygen, the inhibition of a fraction of ATP synthase to promote an increase in the flux of glycolysis, in order to match the higher energetic and biosynthetic demands of proliferation, a phenomenon known as Warburg effect.^33^^,^^34^^,^^35^^,^^40^

The immune system comprehends a heterogeneous population of cells that provide a rapid response to infections and other antigens such as those expressed in transformed cells. At variance with other cells of the immune system, T cells proliferate rapidly upon activation experiencing a metabolic reprogramming quite similar to the Warburg effect, but that it is also accompanied by the biogenesis and functional activity of mitochondria.^41^^,^^42^^,^^43^^,^^44^^,^^45^ Herein, we have asked the question whether IF1 might participate in the commitment of T cells to a particular metabolic phenotype or fate. We demonstrate that upon activation, CD4^+^ T lymphocytes, which in naive state do not express IF1, promote the upregulation of IF1 expression both *in vitro* and *in vivo*. IF1 expression is solely expressed in Th1 but not in Th17 or Treg effector cells. Development of a mouse model with genetic ablation of *Atp5if1* gene (CD4^+^-IF1-KO model) confirmed the primary role of IF1 in gearing the metabolic reprograming of T cells to an enhanced glycolytic phenotype by inhibiting the activity of a fraction of ATP synthase upon activation. Moreover, studies *in vivo* confirmed that CD4^+^-IF1-KO mice have a limited Th1 immune response against bacterial infection compromising mice survival and highlighting the essential role played by IF1 in metabolic reprograming and proliferation of T lymphocytes.

## Results

### Activated but not naive CD4^+^ T lymphocytes express IF1

**Figure 1:**
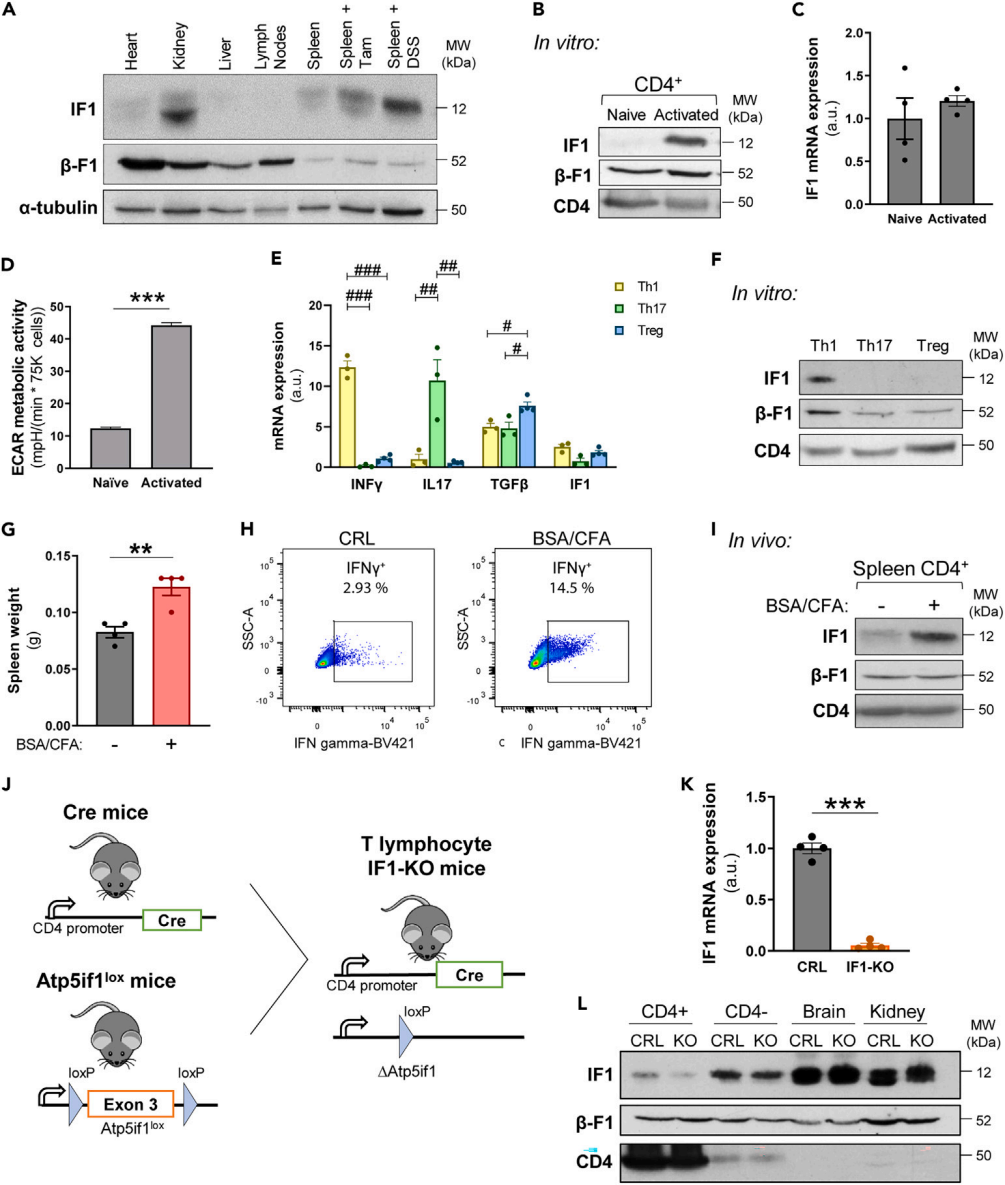
Expression of IF1 in T cells and development of IF1-KO mice in CD4^+^ lymphocytes (A) Representative blot of IF1 and β-F1-ATPase expression in mouse tissue extracts of heart, kidney, liver, lymph nodes and spleen, and in spleen of mice treated with tamoxifen (Tam) or dextran sodium sulfate (DSS). α-tubulin is shown as loading control. (B) Representative blot of IF1 and β-F1-ATPase expression in naive and *in vitro* activated CD4^+^ mouse lymphocytes. CD4 is shown as loading control. (C) Quantification of IF1 mRNA levels in naive and activated CD4^+^ lymphocytes (*n* = 4). (D) Extracellular acidification rates (ECAR) of naive and activated CD4^+^ lymphocytes (*n* = 3). B–D, *In vitro* activation of CD4^+^ lymphocytes was performed by incubating the cells with anti-CD3 and anti-CD28 during 48 h. (E) Quantification of mRNA levels of IFNγ, IL17, TGFβ and IF1 in CD4^+^ lymphocytes polarized to the Th1, Th17 or Treg subsets (n = 3–4). (F) Representative blot of IF1 and β-F1-ATPase expression in CD4^+^ lymphocytes polarized to the Th1, Th17 or Treg subsets. CD4 is shown as loading control. (G) Spleen weight of mice 10 days after immunization with PBS (−) or BSA/CFA (*n* = 4). (H) Representative plots of the percentage of IFNγ^+^ immune cells (CD45^+^) in the spleen of mice 10 days after PBS (−) or BSA/CFA (+) injection. (I) Representative blot of IF1 and β-F1-ATPase expression in CD4^+^ lymphocytes 10 days after PBS (−) or BSA/CFA (+) injection. CD4 is shown as loading control. (J) Schematic of the generation of the CD4^+^-IF1-KO mouse model. IF1-KO mice in CD4^+^ lymphocytes were obtained by breeding CD4-Cre with IF1-floxed mouse lines. (K) Histograms show the quantification of IF1 mRNA levels in CRL and IF1-KO CD4^+^ lymphocytes (*n* = 4). (L) Representative blot of IF1 and β-F1-ATPase expression in extracts of CD4^+^ cells, immune CD4^−^ cells, brain and kidney of CRL and CD4^+^-IF1-KO mice. The histograms show the mean and the error bars ±SEM. ∗∗*p* ≤ 0.01; ∗∗∗*p* ≤ 0.001 when compared to PBS (−) or CRL by Student’s *t* test. #*p* ≤ 0.05; ##*p* ≤ 0.01; ###*p* ≤ 0.001 when compared by one-way ANOVA test and the Tukey multiple comparison test. See also Figure S1.

The role of IF1 in controlling metabolic reprogramming of T lymphocytes is unknown. The expression of IF1 in resting mouse lymph nodes and spleen, where B and T lymphocytes are the major immune populations, is negligible (Figure 1A), suggesting that naive lymphocytes express very low levels of the protein. However, IF1 levels in mouse spleen increase upon treatment with inflammatory agents such as tamoxifen or dextran sulfate sodium (DSS) (Figure 1A), suggesting that IF1 expression is involved in mouse immune responses. Indeed, isolation of CD4^+^ T cells and *in vitro* activation with antibodies against CD3 and CD28 surface antigens confirmed that IF1 is expressed when lymphocytes became activated (Figure 1B). Interestingly, the overexpression of IF1 in activated lymphocytes is controlled at post-transcriptional levels since no changes in cellular IF1-mRNA abundance were observed when compared to naive CD4^+^ lymphocytes (Figure 1C), in agreement with similar findings for the expression of IF1 during cellular differentiation, oncogenesis and tissue-specific expression in mammalian tissues.^32^^,^^39^^,^^46^ Changes in IF1 expression after lymphocyte activation were paralleled by a sharp increase in their rates of glycolysis (Figure 1D).

*In vitro* polarization of naive CD4^+^ lymphocytes to the effector Th1, Th17 and Treg phenotypes (Figure 1E) revealed that only the Th1 subtype expressed IF1 (Figure 1F), despite IF1-mRNA being similarly expressed in the three phenotypes (Figure 1E), further supporting a relevant role for cell-type specific mechanisms in controlling IF1 expression in adaptive immune cells at post-transcriptional levels. Posttranscriptional control of T cell effector functions by aerobic glycolysis has already been reported.^47^

Administration of a BSA/CFA (Bovine Serum Albumin/Complete Freund Adjuvant) mixture to mice elicits a strong immune response as revealed by the sharp increase in spleen weight (Figure 1G) and in IFNγ^+^ producing immune cells (CD45^+^ IFNγ^+^) (Figure 1H) when compared to PBS-treated mice. Congruently with *in vitro* findings (Figure 1B), spleen CD4^+^ lymphocytes of BSA/CFA-treated mice showed a sharp increase in IF1 expression when compared to CD4^+^ lymphocytes of PBS-treated mice (Figure 1I), thus supporting that IF1 expression is also involved in the activation of lymphocytes *in vivo*.

### Development of CD4^+^-IF1-KO mice

To investigate the relevance of IF1 in the immune responses mediated by CD4^+^ T lymphocytes we developed *Atp5if1* knockout mice specifically in T lymphocytes (CD4^+^-IF1-KO) by breeding CD4-Cre mice with IF1-floxed mice^25^ (Figure 1J), to induce the excision of exon 3 and gene inactivation in CD4^+^ expressing cells. Consistently, CD4^+^ lymphocytes derived from CD4^+^-IF1-KO mice revealed negligible expression of IF1 mRNA (Figure 1K) and protein (Figure 1L) when compared to littermates that do not express Cre recombinase, used as controls of the experiments (Figures 1K and 1L). CD4^+^-IF1-KO mice expressed normal levels of IF1 protein in kidney and brain (Figure 1L), stressing the specificity of the genetic inactivation of *Atp5if1* under the CD4 promoter. Only a small reduction in IF1 expression in the CD4^−^ immune fraction was noted (Figure 1L), that might represent CD8^+^ T lymphocytes that also express CD4 during a stage in their development. Depletion of IF1 had no relevant effect on the expression of its target protein, the β-subunit of the mitochondrial ATP synthase (Figure 1L).

### Non-stressed CD4^+^-IF1-KO mice show no alterations in longevity, or in metabolic and hematologic parameters

Male and female IF1-KO mice showed no alteration in body weight or lifespan when compared to control IF1-expressing littermates (Figures S1A, and S1B). Likewise, the glucose tolerance test in male IF1-KO mice yielded comparable results to the controls (Figure S1C). Hematologic analyses of IF1-KO mice showed normal levels of circulating basophils (BAS), neutrophils (NEU), eosinophils (EOS), lymphocytes (LYM), and monocytes (MON) (Figure S1D), and erythrocytes and platelets (Figure S1E). Likewise, blood hemoglobin concentration (HGB) and the mean corpuscular hemoglobin concentration (MCHC) were the same as in controls (Figure S1E).

Flow cytometry analyses of the different populations of T lymphocytes in lymphoid organs of CD4^+^-IF1-KO mice revealed no relevant differences in cell counts when compared to control mice (Figure S1F). Likewise, the content of the main effector subtypes both in spleen and lymph nodes (Figure S1G), showed no differences in aseptic conditions,^48^^,^^49^^,^^50^ thus supporting that IF1 is not required for the development and maturation of T lymphocytes in the thymus and hence, does not compromise hematopoiesis. The same observation has been reported when T lymphocytes have a deletion of mitochondrial *Tfam*^43^ or *Cox10*.^44^ Altogether, these findings indicate that the bioenergetic function of mitochondria is dispensable for the generation of naive T lymphocytes.

### CD4^+^ IF1-KO lymphocytes differentiate mitochondria upon activation

**Figure 2:**
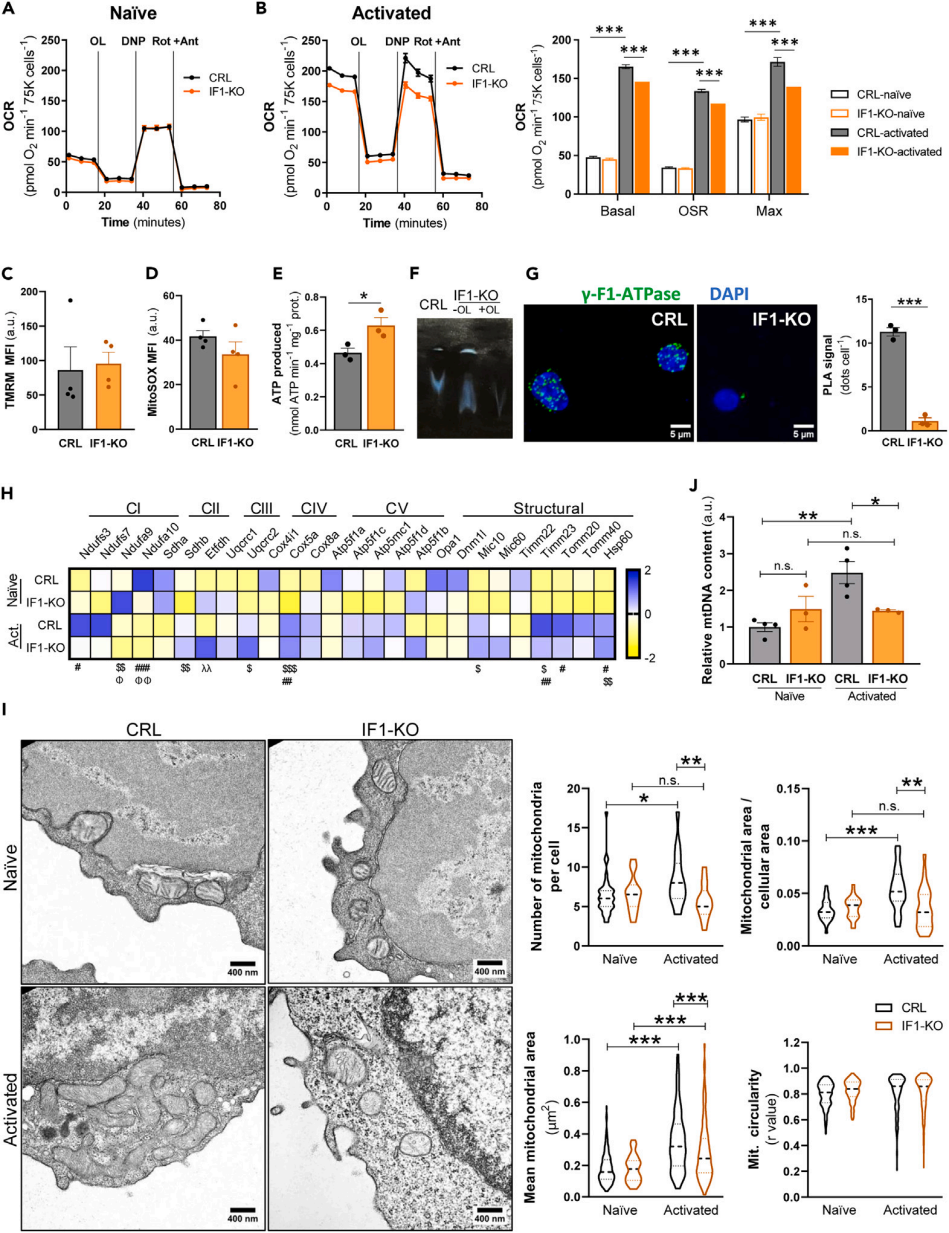
The biogenesis of mitochondria is restrained in activated CD4^+^ IF1-KO lymphocytes (A and B) Profiles of oxygen consumption rates (OCR) of naive (a) (*n* = 3) and activated (b) (*n* = 3) murine CRL and IF1-KO CD4^+^ lymphocytes using glucose as respiratory substrate. The addition of oligomycin (OL), 2,4-dinitrophenol (DNP) and rotenone (Rot) plus antimycin A (Ant) is indicated. (B) Histogram shows the quantification of basal, oligomycin-sensitive (OSR) and maximal respiration rates of CRL and IF1-KO naive and activated CD4^+^ lymphocytes. (C) Mitochondrial membrane potential in CRL and IF1-KO activated CD4^+^ lymphocytes (*n* = 4). MFI, mean fluorescent intensity. (D) Mitochondrial reactive oxygen species (mtROS) production in CRL and IF1-KO activated CD4^+^ lymphocytes (*n* = 4). (E) Histogram shows the rate of ATP production per mg of cellular protein in digitonin-permeabilized CRL and IF1-KO activated CD4^+^ lymphocytes (*n* = 3). (F) Representative ATP hydrolytic activity of complex V in CN-PAGE gels of CRL and IF1-KO activated CD4^+^ lymphocytes. Where indicated, 2 μM oligomycin (OL) was added in the sample to inhibit ATP hydrolysis. (G) Representative images of Proximity Ligation Assays (PLA) using γ-F1-ATPase as target (green dots) in CRL and IF1-KO activated CD4^+^ lymphocytes. DAPI (blue) stained nuclei. Histograms show the number of PLA signals per cell (*n* = 3). (H) Heatmap shows normalized Z-scores of mRNA levels of genes encoding different mitochondrial proteins in naive and activated CRL and IF1-KO CD4^+^ lymphocytes (n = 3–4). #*p* ≤ 0.05; ##*p* ≤ 0.01; ###*p* ≤ 0.001 when compared naive CRL vs. activated CRL; $*p* ≤ 0.05; $$*p* ≤ 0.01; $$$*p* ≤ 0.001 when compared naive CRL vs. naive IF1-KO; λλp ≤0.01; when compared activated CRL vs. activated IF1-KO; Φp ≤ 0.05; ΦΦp ≤0.01 when compared naive IF1-KO vs. activated IF1-KO by one-way ANOVA and Tukey multiple comparison test. (I) Representative electron micrographs of naive (upper panels) and activated (lower panels) CRL and IF1-KO CD4^+^ lymphocytes. Violin plots show the number of mitochondria per cell (*n* = 17–36), the mitochondrial area per cytoplasmatic surface (*n* = 17–28), the mean mitochondrial area (*n* = 59–256) and the mean mitochondrial circularity (*n* = 59–256) assessed in micrographs. (J) mtDNA copy number analysis in naive and activated CRL or IF1-KO CD4^+^ lymphocytes (n = 3–4). *In vitro* activation of CD4^+^ lymphocytes was performed by incubating the cells with anti-CD3 and anti-CD28 during 48 h. The profiles and histograms show the mean and the error bars ±SEM. n.s., no significant. ∗*p* ≤ 0.05; ∗∗∗*p* ≤ 0.001 when compared to CRL or naive by Student’s *t* test.

The energetic demand of naive CD4^+^ lymphocytes is very low.^48^^,^^49^^,^^50^ Consistently, cellular respiration in naive control (CRL) and IF1-KO CD4^+^ lymphocytes was very low and not significantly different between the two genotypes (Figures 2A and 2B). However, upon activation, both genotypes of CD4^+^ lymphocytes showed a sharp increase (∼4-fold) in cellular respiration (Figure 2B), although of lesser intensity for IF1-KO lymphocytes (Figure 2B), supporting that the absence of IF1 does not largely affect the formation of functionally active mitochondria (i.e., functional differentiation) upon T cell activation.^42^^,^^43^^,^^44^ The differentiation of pre-existing mitochondria has also been reported in rat liver shortly after birth^51^^,^^52^ and during differentiation of mesenchymal stem cells into osteocytes.^39^ Interestingly, CD4^+^ CRL and IF1-KO lymphocytes showed no relevant differences in mitochondrial membrane potential (ΔΨm) (Figure 2C) or mtROS production (Figure 2D), in agreement with the subtle differences observed in the rates of respiration between both genotypes after activation (Figure 2B).

Determination of both catalytic activities of the mitochondrial ATP synthase, the oligomycin-sensitive ATP synthesis (Figure 2E) and ATP hydrolysis (Figure 2F), in permeabilized activated lymphocytes showed increased rates in IF1-KO cells when compared to CRL cells, indicating that the inhibition of a fraction of the enzyme was relieved due to the absence of IF1. Interestingly, IF1-KO activated lymphocytes show lower oligomycin-sensitive respiration when compared to the controls (Figure 2B), but higher ATP synthetic activity (Figure 2E). This difference most likely results from how mitochondria are energized in each assay. Moreover, because in the determination of the ATP production rate the ATP synthase activity is assessed at the maximal rate achievable by the enzyme at saturating conditions. Remarkably, ablation of IF1 resulted in the loss of oligomeric IF1-inhibited assemblies of ATP synthase (Figure 2G), as assessed by Proximity Ligation Assays using the γ-subunit of ATP synthase as target.^28^ These results (Figures 2E–2G), indicate that mitochondria of activated CD4^+^ lymphocytes, as in other cellular types that express IF1,^25^^,^^26^^,^^27^^,^^28^ also contain a fraction of IF1-inhibited ATP synthase.

### The biogenesis of mitochondria is restrained in activated CD4^+^ IF1-KO lymphocytes

In addition to the functional differentiation of mitochondria (Figures 2A and 2B), lymphocytic activation requires the biogenesis of new mitochondria to increase the number of organelles per cell.^42^ Transcriptomic analysis of OXPHOS and structural mitochondrial proteins in naive CRL and IF1-KO lymphocytes revealed marginal differences between them (Figure 2H), in agreement with the lack of differences in respiratory rates (Figure 2A). However, the same analysis between naive and activated CD4^+^ lymphocytes revealed a sharp increase in the expression of several genes of respiratory complexes and structural proteins of the organelle (NDUFS3, NDUFS7, HSP60, TIM and TOM) (Figure 2H), a finding compatible with a more active biogenesis of mitochondria upon the activation of lymphocytes. Interestingly, we observed minor differences in gene expression between activated IF1-KO and CRL lymphocytes (Figure 2H), in agreement with minor changes in the rates of respiration (Figures 2A and 2B), ΔΨm (Figure 2C) and mtROS production (Figure 2D). The marginal differences in gene expression and functional activity of mitochondria between both genotypes of activated lymphocytes support that the differentiation of mitochondria (Figures 2A and 2B) is primarily exerted by mechanisms of translational control, in agreement with previous similar observations in the differentiation of mitochondria of human mesenchymal stem cells,^39^ rat liver mitochondria soon after birth^51^^,^^52^ and the relevance of posttranscriptional control in T cell effector functions.^47^

Electron microscopy analysis of naive and activated CRL and IF1-KO CD4^+^ lymphocytes revealed sharp differences in mitochondrial parameters upon lymphocyte activation (Figure 2I). We observed a higher increase in the number of organelles, in the cellular area occupied by mitochondria and in the overall area of mitochondria in CRL when compared to IF1-KO lymphocytes (Figure 2I), supporting that the biogenesis of mitochondria upon cellular activation is blunted in IF1-KO lymphocytes (Figure 2I). However, and despite the large differences in mitochondrial counts between CRL and IF1-KO lymphocytes, we did not observe significant differences in the structure or overall shape of the organelle between CRL and IF1-ablated T lymphocytes (Figure 2I).

Determination of mtDNA copy number upon lymphocyte activation confirmed the arrested biogenesis of mitochondria in IF1-KO lymphocytes (Figure 2J). In fact, whereas activated CRL lymphocytes showed a 2-fold increase in mtDNA content (Figure 2J), IF1-KO lymphocytes displayed no increase in mtDNA (Figure 2J), thus supporting that the sharp rise experienced in mitochondrial respiration upon activation in both genotypes (Figures 2A and 2B) represents an event of differentiation of pre-existing organelles.^39^^,^^51^^,^^52^

### Genetic ablation of IF1 in CD4^+^ T lymphocytes limits aerobic glycolysis and cellular proliferation

**Figure 3:**
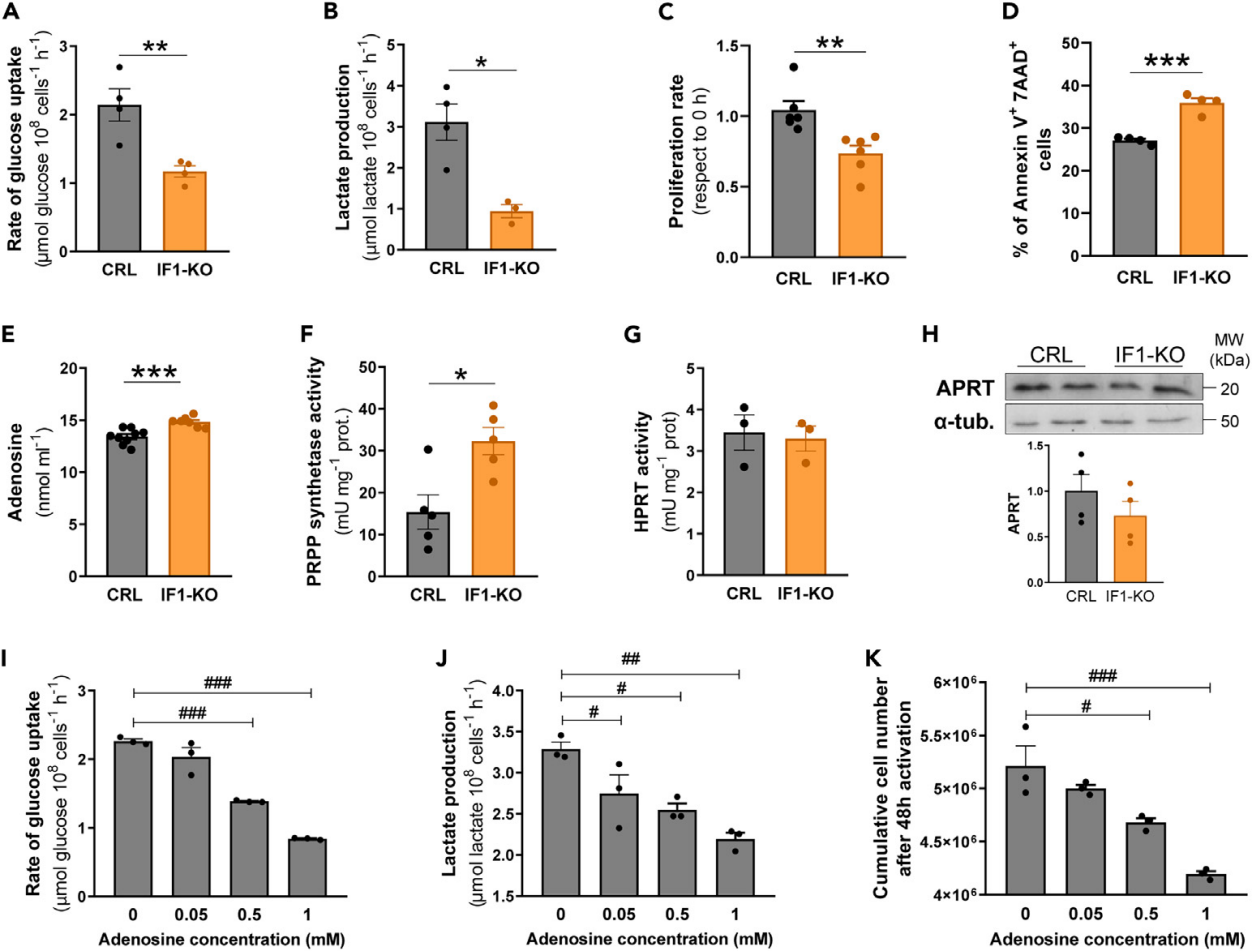
IF1-KO lymphocytes have restrained rates of glycolysis and proliferation upon activation (A) Rates of glucose uptake in CRL and IF1-KO activated CD4^+^ lymphocytes (*n* = 4). (B) Rates of lactate production in CRL (*n* = 4) and IF1-KO (*n* = 3) activated CD4^+^ lymphocytes. (C) Proliferation rates of CRL and IF1-KO CD4^+^ lymphocytes after 24 h of activation (*n* = 6). (D) Percentage of dead (AnnexinV^+^/7AAD^+^) CRL and IF1-KO CD4^+^ lymphocytes after 48 h of activation (*n* = 4). (E) Determination of adenosine released into the culture medium of CRL (*n* = 9) and IF1-KO (*n* = 7) CD4^+^ lymphocytes after 48 h of activation. (F and G) Activities of phosphoribosyl pyrophosphate synthetase (PRPP synthetase) (f) (*n* = 5) and hypoxanthine-guanine phosphoribosyl transferase (HPRT) (*n* = 3) (k) in CRL and IF1-KO activated CD4^+^ lymphocytes. (H) Representative blot of the expression of adenine phosphoribosyl transferase (APRT) in CRL and IF1-KO activated CD4^+^ lymphocytes. α-tubulin is shown as loading control. Histogram shows the expression level relative to α-tubulin (*n* = 4). (I–K) Rates of glucose uptake (i), lactate production (j) and proliferation (k) in CRL CD4^+^ lymphocytes treated with the indicated doses of adenosine during activation (*n* = 3). *In vitro* activation of CD4^+^ lymphocytes was performed by incubating the cells with anti-CD3 and anti-CD28 during 48 h. The histograms show the mean and the error bars ±SEM. ∗*p* ≤ 0.05; ∗∗*p* ≤ 0.01; ∗∗∗*p* ≤ 0.001 when compared to CRL by Student’s *t* test. #*p* ≤ 0.05; ##*p* ≤ 0.01; ###*p* ≤ 0.001 when compared by one-way ANOVA and Tukey multiple comparison test.

Activation of aerobic glycolysis is required to provide the carbon skeletons and energy that are needed to promote the accretion of cellular mass for the biogenesis of mitochondria and cellular proliferation.^33^^,^^34^^,^^35^ Interestingly, we observed that the glucose uptake rates in activated IF1-KO lymphocytes were around half when compared to CRL cells (Figure 3A). This finding was also paralleled by a 3-fold lower rate of lactate production in IF1-KO lymphocytes with respect to CRL lymphocytes (Figure 3B). These results strongly support that activated CD4^+^ IF1-KO lymphocytes have a severe restriction in the metabolic flux through glycolysis, what may limit the availability of building blocks for the biogenesis of mitochondria (Figures 2I and 2J), thereby compromising lymphocytic proliferation in response to activation. Indeed, IF1-KO lymphocytes proliferate less than CRL upon activation (Figure 3C) and show increased rates of cell death when compared to controls (Figure 3D).

Recent findings indicate that ablation of IF1 in the intestinal epithelium promotes mitochondrial ATP degradation by a futile cycle that arises when eliminating the inhibitor of ATP synthase.^27^ In this situation, both cancer cells and the serum of IF1-ablated mice accumulated adenosine,^27^ product of the catabolism of ATP. Moreover, futile ATP hydrolysis promoted the activation of *de novo* purine biosynthesis and salvage pathways in the intestine and in cancer cells.^27^ Consistent with these findings, we observed that IF1-KO lymphocytes released more adenosine to the culture medium (Figure 3E), and showed higher activity of phosphoribosyl-pyrophosphate synthetase, the rate-limiting enzyme of purine biosynthesis (Figure 3F), when compared to CRL cells (Figures 3E and 3F). Altogether, these results support the activation of purine biosynthesis as the likely result of ATP hydrolysis in mitochondria of IF1-KO lymphocytes. At variance with the findings in colon, that indicated that ablation of IF1 also promoted the activation of purine salvage pathways,^27^ we observed that neither the activity of hypoxanthine-guanine phosphoribosyl transferase (Figure 3G) nor the expression of adenine phosphoribosyl transferase (Figure 3H), were significantly different between the two genotypes, thus supporting no activation of the purine salvage pathways in IF1-KO lymphocytes.

Adenosine inhibits the activation and function of CD4^+^ and CD8^+^ T lymphocytes.^53^^,^^54^^,^^55^^,^^56^ Consistently, we observed that adenosine restrained, in a dose-dependent manner, the rates of glucose uptake (Figure 3I), lactate production (Figure 3J) and cellular proliferation (Figure 3K) upon activation in CRL CD4^+^ lymphocytes, suggesting that an uncontrolled ATP hydrolysis by uninhibited mitochondrial ATP synthase, due to the lack of IF1, could also contribute to an adenosine-mediated block in the activation of CD4^+^ lymphocytes.

### Absence of IF1 in CD4^+^ T lymphocytes compromises survival upon bacterial infection *in vivo*

An impaired *in vitro* activation of CD4^+^ IF1-KO lymphocytes raises the question of the *in vivo* relevance of IF1, when mice are confronted to stimuli that induce immune responses. LPS-induced endotoxemia in mice triggers an acute inflammatory response that mimics in several aspects a Gram negative bacterial infection, but without the possibility of generating true infection.^57^ To this end, we administered LPS to control and IF1-KO mice, in a dose previously calculated to promote 25% lethality in control mice. No differences were observed in mouse survival between both genotypes (Figure S2A) or in the circulating levels of the pro-inflammatory cytokines IFNγ and IL-1β in the acute phase of inflammation (Figure S2B), most likely because the response to LPS is extremely fast, and hence primarily dominated by the innate immune system.^57^

**Figure 4:**
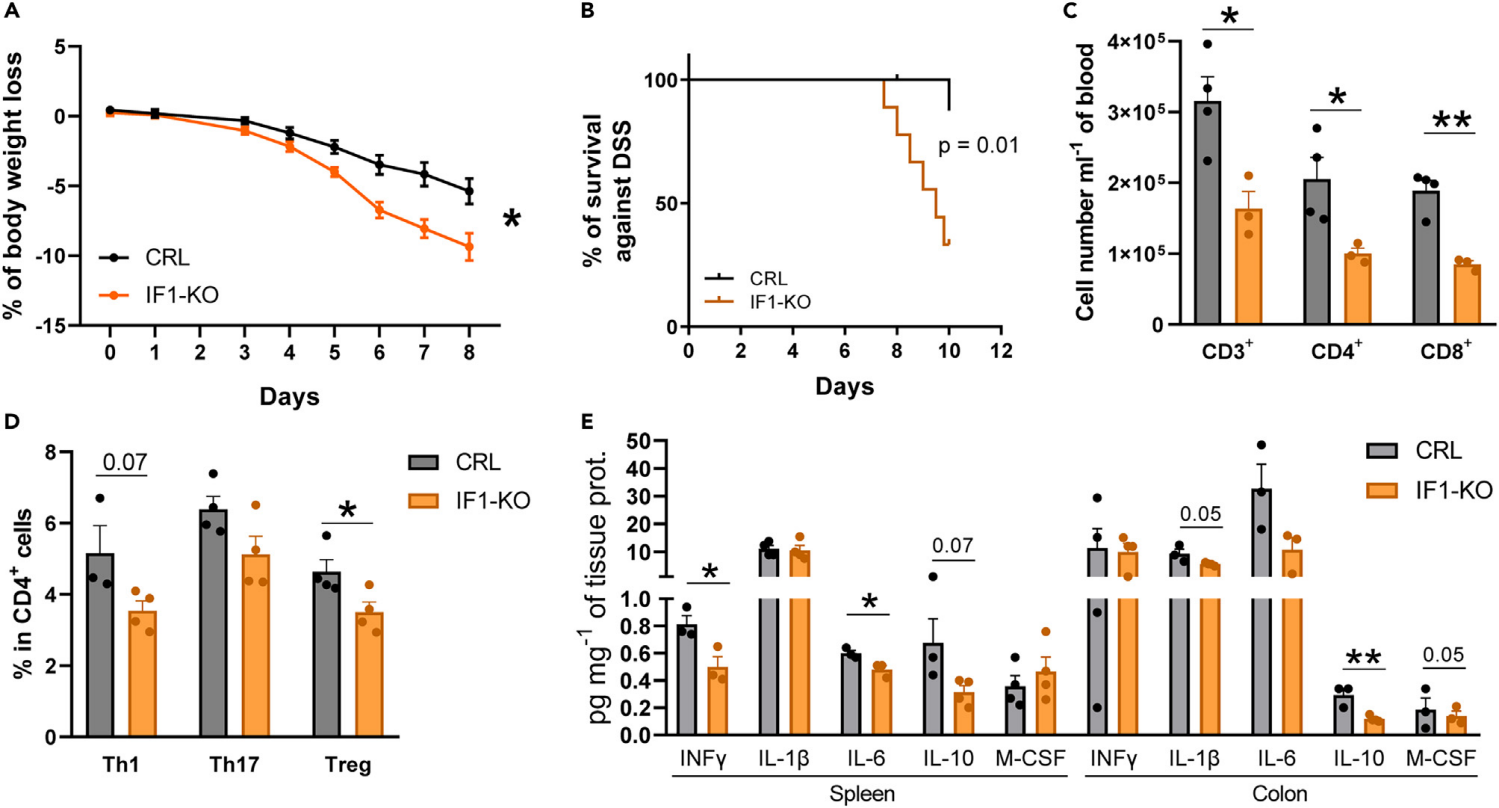
Survival of CD4^+^-IF1-KO mice is compromised in response to bacterial infection due to a restrained adaptive immune response (A) Progressive weight change of CRL (*n* = 5) and IF1-KO (*n* = 4) mice treated with 2.5% dextran sodium sulfate (DSS) in the drinking water for nine days. (B) Kaplan–Meier survival analysis of CRL (*n* = 9) and IF1-KO mice (*n* = 9) after treatment with 2.5% DSS in the drinking water. The *p*-value of the log rank test is shown. (C) Number of CD3^+^ (CD3^+^;CD45^+^;DAPI^−^), CD4^+^ (CD4^+^;CD45^+^;DAPI^−^) and CD8^+^ (CD8^+^;CD45^+^;DAPI^−^) cells in the blood of CRL and IF1-KO mice after 6-day treatment with 2.5% DSS (n = 3–4). (D) Percentage of Th1 (INFγ^+^;CD4^+^;CD45^+^), Th17 (IL17^+^;CD4^+^;CD45^+^) and Treg (FoxP3^+^;CD4^+^;CD45^+^) cells in the blood of CRL and IF1-KO mice after 6-day treatment with 2.5% DSS (n = 3–4). (E) Multiplexed quantitative analysis of cytokines and chemokines. Histograms show the mean and the error bars ±SEM of cytokine levels in the spleen and colon of CRL and IF1-KO mice after 6-day treatment with 2.5% DSS (n = 3–4). ∗*p* ≤ 0.05; ∗∗*p* ≤ 0.01 when compared to CRL by Student’s *t* test. See also Table S1.

To generate an inflammatory response that allowed the activation of the adaptive immune system we administered non-lethal doses of DSS in the drinking water of the animals for nine days.^27^^,^^58^ DSS promotes an inflammatory response in the intestinal barrier that allows the invasion of bacteria into the circulation, creating a real infection that engages both the innate and adaptive responses.^27^^,^^59^^,^^60^ In this situation, IF1-KO mice showed an aggravated weight loss when compared to littermate controls (Figure 4A), indicating a worsening permeability barrier. Remarkably, whereas 90% of control mice tolerated the DSS-treatment only 33% of IF1-KO mice survived (Figure 4B), supporting the idea that the CD4^+^ guided adaptive immune response is compromised in IF1-KO mice upon a bacterial infection. This idea was further confirmed by the diminished circulating levels of T lymphocytes (Figure 4C) and in the lesser polarization of CD4^+^ lymphocytes to Th1 and Treg subsets in CD4^+^-IF1-KO mice (Figure 4D).

Multiplex analysis of spleen cytokines (Table S1) revealed a reduction in the pro-inflammatory cytokines INFγ and IL-6 and in the anti-inflammatory cytokine IL-10 in CD4^+^-IF1-KO mice (Figure 4E) in response to DSS. Similarly, cytokine analysis in the colon (Table S1) also showed a reduced content of IL1α, IL-6, IL-10 and M-CSF in IF1-KO mice (Figure 4E). Altogether, the cytokine analysis also supports a diminished immune response upon DSS-induced bacterial infection. Unexpectedly, cytokine analysis in serum of IF1-KO mice revealed no significant differences with the exception of an increase in the pro-inflammatory cytokine INFγ (Table S1), which may reveal the stressful phenotype resulting from the genetic ablation of IF1. Overall, the results support that upon bacterial infection, CD4^+^ lymphocytes devoid of IF1 cannot proliferate and polarize properly to the different effector subsets *in vivo*, hence compromising the antibacterial immune response what results in increased mortality.

## Discussion

Herein, we report that IF1, the physiological inhibitor of the mitochondrial ATP synthase, is not expressed in naive T lymphocytes. Only upon the activation of T lymphocytes IF1 is overexpressed paralleling a sharp increase in metabolic activity. Moreover, IF1 is overexpressed in T lymphocytes polarized to the Th1 subset, which guide pro-inflammatory responses against a broad range of antigens. The upregulation of IF1 upon T cell activation and polarization to the Th1 subset, in the absence of relevant changes in mRNA abundance, is likely due to protein stabilization. In fact, and at variance with other subunits of the ATP synthase,^61^ IF1 has a very rapid turnover rate (∼100 min).^32^ The accumulation of IF1 when cells are treated with inhibitors of serine proteases^32^^,^^39^ or metalloproteases^62^ suggest that members of these families are involved in the cell-type specific degradation of IF1 through complex processes in which different proteases may play complementary roles.^39^ In any case, the stabilization of IF1 protein appears to be a relevant mechanism in the maintenance of stemness in human mesenchymal stem cells^39^ and in the activation of T lymphocytes that deserve future specific investigations.

The genetic ablation of IF1 in T lymphocytes impairs their metabolism and thus affects their proliferation upon activation and Th1-mediated responses, thereby compromising mouse survival after a bacterial infection. Activated lymphocytes lacking IF1 show an increase in ATP synthase and hydrolase activities, similar to those reported in Jurkat and other IF1-KO cancer cells^28^ and in brain, colon, kidney and heart mitochondria of IF1-KO mice,^25^^,^^27^^,^^28^ thus supporting that activated lymphocytes have a fraction of ATP synthase inhibited by IF1. Remarkably, genetic ablation of IF1 in T lymphocytes prevents the activation of glucose uptake and its utilization in aerobic glycolysis, emphasizing the role of the IF1-inhibited pool of ATP synthase in governing the reprogramming of cellular metabolism to an effective glycolytic flux that supplies the metabolic precursors for the biogenesis of mitochondria and cellular proliferation. These findings agree with other studies reporting that the inhibition of ATP synthase is a key event in the induction of glycolysis in CD4^+^ T lymphocytes and potentiates a Th1 response against malaria parasite.^63^ Moreover, our findings align well with the role described for IF1 in cancer^17^^,^^29^^,^^32^^,^^64^^,^^65^ and in the reprogramming of somatic cells into iPSC and in the maintenance of stemness.^36^^,^^37^^,^^39^ However, the inhibition of ATP synthesis by IF1 is also known to promote the generation of mtROS, as a result of mitochondrial hyperpolarization and reduction of the electron flow through the respiratory chain.^29^ The IF1-mediated increase in mtROS production is mild and contributes to rewiring nuclear transcriptional programs^29^ that signal different mitohormetic programs at the cellular and organismal levels.^15^^,^^25^^,^^58^ Hence, we cannot exclude the additional participation of IF1 in mediating the mtROS signal involved in the transcriptional mechanisms that contribute to T cell activation.^42^^,^^66^^,^^67^

Consistent with the large differences in metabolic activities that exist between Th1, Th17 and the highly OXPHOS dependent Treg effector subtypes,^68^^,^^69^^,^^70^ we observed that only Th1 lymphocytes express IF1. Th1 and Th17 lymphocytes are highly glycolytic cells. However, Th1 cells also depend heavily on OXPHOS.^42^^,^^66^^,^^71^ Notably, the expression of IF1 in activated lymphocytes parallels the sharp increase in the biogenesis and functional differentiation of mitochondria. This might explain the requirement for the expression of IF1 in Th1 lymphocytes, in order to exert partial control on the activity of ATP synthase, to balance the flux of glycolysis required for proliferation.

The inhibition of the ATP synthetic activity of ATP synthase by IF1 during active OXPHOS conditions is debated. Single molecule studies of the IF1-inhibited F1 complex showed that IF1 is ejected from ATP synthase when the Fo rotor is forced to rotate clockwise for the synthesis of ATP.^72^ These findings,^72^ together with the pH-regulated activity of IF1,^2^^,^^21^^,^^22^^,^^23^ and the observed inhibition of the ATP hydrolytic activity of ATP synthase upon mitochondrial de-energization,^3^^,^^18^ provided strong support to the idea that IF1 behaves only as a unidirectional inhibitor that prevents ATP hydrolysis in mitochondria.^2^ However, these findings excluded the idea that under normal OXPHOS conditions a fraction of IF1 could bind and inhibit a pool of ATP synthase in the mitochondrion. Perhaps, because the bioenergetic function of the mitochondrion was not considered to be compartmentalized in different cristae and/or other microdomains of the IMM. However, the coexistence of active and inactive ATP synthase in the IMM contributes to explain the heterogeneous distribution of the ΔΨm throughout different cristae of the organelle,^28^^,^^73^^,^^74^ supporting their bioenergetic independence. Moreover, this notion agrees with results in cellular and mouse models of loss and gain of function of IF1 that have provided evidence that the mitochondrion contains a fraction of IF1-bound and inhibited ATP synthase under normal OXPHOS conditions.^24^^,^^25^^,^^26^^,^^27^^,^^28^ In fact, IF1 has been recently shown to interact with ATP synthase at the local level in the mitochondrion, establishing the heterogeneity of ΔΨm in the IMM, where cristae microdomains of high ΔΨm co-distribute with high levels of IF1 bound to the enzyme.^28^ Additionally, the recent identification of a specific inhibitor of the ATP hydrolase activity of the enzyme,^75^ and the cryo-EM structures of IF1-bound and inhibited ATP synthase isolated from mammalian hearts,^9^^,^^76^ further support that the mitochondrion contains a fraction of IF1-inhibited enzyme under OXPHOS conditions.

In this context, IF1 is not only playing a regulatory role on the ATP synthase/hydrolase activities, but also serves as a structural element involved in the oligomerization of the enzyme, and hence, in mitochondrial cristae structure of some tissues.^4^^,^^28^^,^^76^ Indeed, we show that the mitochondrial amount of IF1 in T cells largely correlates with the content of oligomeric assemblies of the enzyme, as recently reported in cancer cells and in isolated tissue mitochondria of global IF1-KO mice,^28^ and previously described in different cellular types by other approaches.^3^^,^^25^^,^^26^^,^^77^^,^^78^ However, although ablation of IF1 highly reduced the formation of oligomers of ATP synthase in activated lymphocytes, it did not alter cristae structure, as we have reported previously in other types of differentiated cells (neurons and colonocytes) that do express high levels of IF1.^25^^,^^27^^,^^28^ The reason for this observation stands for scrutiny and might stem from cell-type specific differences in the proteinaceous determinants of the IMM, since mitochondria in liver and skeletal muscle, which are tissues devoid of IF1,^28^^,^^46^ do have well-developed cristae.

Remarkably, CD4^+^-IF1-KO mice showed no differential response to LPS-induced endotoxemia when compared to control mice. Endotoxemia triggers an innate immune response less intense than an actual infection.^79^ In contrasts, DSS promotes the invasion of intestinal tissue by bacteria and a Th1/Th17 adaptive immune response due to an increase in the permeability of the intestinal barrier.^27^ When mice were confronted to DSS-induced inflammation, most IF1-KO mice died because the lack of IF1 blunted *in vivo* the T lymphocyte adaptive immune response by limiting proliferation of Th1 cells. These findings strongly support that IF1-mediated regulation of the ATP synthase lies at the center of mitochondrial biogenesis and proliferation of T lymphocytes by controlling glucose uptake and its utilization in aerobic glycolysis. Indeed, we propose a mechanism whereby IF1 controls the activity of the ATP synthase in activated T lymphocytes and may be key for the induction of glycolysis and Th1 effector functions in the context of infection.^63^

Ablation of IF1 in mouse colonocytes increases the rate of mitochondrial ATP hydrolysis, as a result of abolishing the pool of inhibited enzyme.^27^ Moreover, the absence of IF1 promotes the accumulation of adenosine in the culture medium and the activation of *de novo* purine biosynthesis to compensate for the degradation of ATP.^27^ Consistently, we observed that deletion of IF1 in T cells also promotes the accumulation adenosine and the activation PRPP synthetase. Although we only observed a small accumulation of adenosine in cell cultures, we suggest that *in vivo* the local concentration of adenosine in secondary lymphoid organs and targeted tissues could represent an additional mechanism to repress the adaptive immune response in CD4^+^-IF1-KO mice.

Overall, our findings stress that functionality of T cells is governed by their metabolism and indicate the relevance of the IF1-ATP synthase axis in the metabolic reprogramming required for an efficient adaptive immune response.

### Limitations of the study

We elucidated that the expression of IF1 is required to inhibit a fraction of ATP synthase in mitochondria of CD4^+^ T lymphocytes in order to promote the metabolic reprogramming to an enhanced glycolytic phenotype that supports the biogenesis of mitochondria and cellular proliferation after activation and polarization of lymphocytes to the Th1 subset. However, the post-transcriptional mechanisms that control the cell-type specific upregulation of IF1 in CD4^+^ lymphocytes remain unknown. We generated and used mice devoid of IF1 in T lymphocytes and a DSS model of colon inflammation to illustrate that IF1-knockout mice cannot mount an effective CD4^+^ immune response against bacterial infection what compromises their survival. Nonetheless, due to the limitations inherent in the present proof-of-concept study, further research is essential to clarify the role of IF1 in guiding pro-inflammatory responses against infection and anti-tumor immunity as well as its potential as a target for therapy.

## STAR★Methods

### Key resources table

**Table:** 

REAGENT or RESOURCE	SOURCE	IDENTIFIER
**Antibodies**		

Mouse anti-α-tubulin (clone DM1A)	MilliporeSigma	Cat# T9026; RRID: AB_477593
Mouse anti-Cd11b PerCP-Cy5.5	Invitrogen	Cat# 45-0112-82; RRID: AB_953558
Mouse anti-CD3 Pe-Cy7	Invitrogen	Cat# 25-0031-82; RRID: AB_469572
Mouse anti-CD45 APC	Invitrogen	Cat# 17-0451-82; RRID: AB_469392
Mouse anti-CD8 APC/Cy7	BioLegend	Cat# 100713; RRID: AB_312752
Mouse anti-FOXP3 PE	Invitrogen	Cat# 12-5773-80; RRID: AB_465935
Mouse anti-IFNγ BV421	BioLegend	Cat# 505830; RRID: AB_2563105
Mouse anti-IL-17 APC	Invitrogen	Cat# 17-7177-81; RRID: AB_763580
Mouse anti-Ly-6C FITC	BioLegend	Cat# 128005; RRID: AB_1186134
Mouse anti-γ-F1-ATPasa	José M. Cuezva	Willers et al.^80^
Peroxidase-conjugated goat anti-rabbit IgGs (1:5,000)	Nordic Immunology	Cat# GAR/IgG(H + L)/PO
Peroxidase-conjugated rabbit anti-mouse IgGs (1:5,000)	Nordic Immunology	Cat# RAM/IgG(H + L)/PO
Rabbit anti-β-F1-ATPasa	José M. Cuezva	Cuezva et al.^40^
Rabbit anti-APRT	ABclonal	Cat# A5456; RRID. AB_2766257
Rabbit anti-CD4 (clone EPR19514)	Abcam	Cat# ab183685; RRID: AB_2686917
Rabbit anti-CD4 FITC	Invitrogen	Cat# 11-0042-82; RRID: AB_464896
Rabbit anti-IF1	José M. Cuezva	Esparza-Molto et al.^46^
Rat anti-CD3 (clone 17A2)	Invitrogen	Cat# 16-0032-82; RRID: AB_468851
Rat anti-F4/80 PE	Bio-Mad	Cat# MCA497RT
Rat anti-IFNγ (clone XMG1.2)	Invitrogen	Cat# 14-7311-81; RRID: AB_468467
Rat anti-IL-4 APC	Invitrogen	Cat# 17-7041-81; RRID: AB_469493
Rat anti-IL4 (clone 11B11)	Invitrogen	Cat# 14-7041-81; RRID: AB_468410
Syrian hamster anti-CD28 (clone 37.51)	Invitrogen	Cat# 16-0281-82; RRID: AB_468921

**Chemicals, peptides, and recombinant proteins**		

2,4-Dinitrophenol	MilliporeSigma	Cat# D198501
40% Acrylamide solution	Bio-Rad	Cat# 1610140
Adenosine	MilliporeSigma	Cat# A9251
ADP	MilliporeSigma	Cat# A2754
Antimycin A	MilliporeSigma	Cat# A8474
ATP	MilliporeSigma	Cat# A2383
Bio-Rad Protein Assay	Bio-Rad	Cat# 5000001
Bovine Serum Albumin (BSA)	Nzytech	Cat# MB04602
Brefaldin A Solution	eBioscience	Cat# 00-4506-51
Carbonyl cyanide 4-(trifluoromethoxy)phenylhydrazone (FCCP)	MilliporeSigma	Cat# C2920
CellTrace^TM^ Violet	Life Technologies	Cat# C34557
Complete Mini EDTA-free protease inhibitor cocktail	MilliporeSigma	Cat# 11836170001
D-Luciferin	Invitrogen	Cat# L2916
DAPI	Merck KGaA	Cat# 268298
Dextran Sulfate 40 Sodium Salt	PanReac AppliChem	Cat# A3261,0250
Digitonin	MilliporeSigma	Cat# D5628
Epoxy Resin Epon 812	TAAB Laboratories	Cat# E202
Fetal Bovine Serum (FBS)	MilliporeSigma	Cat# F7524
Ionomycin	Invitrogen	Cat# I24222
L-Lactate Dehydrogenase (L-LDH)	MilliporeSigma	Cat# 10127230001
Lipopolysaccharide (LPS)	Sigma-Aldrich	Cat# L4516
Luciferase from *Photinus pyralis*	Merck KGaA	Cat# SRE-0045
MitoSOX	Invitrogen	Cat# M36008
Murine IL-12 p70	PeproTech	Cat# 210-12
Murine IL-2	PeproTech	Cat# 212-12
Murine IL-23	R & D Systems	Cat# 1887-ML
Murine IL-6	PeproTech	Cat# 216-16
Murine TGF-β	R & D Systems	Cat# 7666-MB
NADH	MilliporeSigma	Cat# N8129
Oligomycin	MilliporeSigma	Cat# O4876
P,^1^P^5^-di(adenosine-5′) pentaphosphate	MilliporeSigma	Cat# D4022
Paraformaldehyde 4% (PFA)	Santa Cruz Biotechnology	Cat# 30525-89-4
Phorbol 12-myristate 13-acetate (PMA)	Sigma-Aldrich	Cat# P1585
Phosphatase inhibitor cocktail-2	MilliporeSigma	Cat# P5726
Phosphoenol-pyruvate	MilliporeSigma	Cat# 10108294001
Poli-L-Lysine	Sigma-Aldrich	Cat# P8920
Ponceau Red	MilliporeSigma	Cat# P7170
Pyruvate kinase (PK)	MilliporeSigma	Cat# 10128155001
Rotenone	MilliporeSigma	Cat# R8875
Succinate	MilliporeSigma	Cat# S7501
Tetramethylrhodamine Methyl Ester (TMRM)	Invitrogen	Cat# T668
Tissue Protein Extraction Reagent (T-PER)	Thermo Fisher	Cat# 78510
Triton X-100	Merck KGaA	Cat# 9030-19-5
Trizol	Invitrogen	Cat#15596026
Tween 20	EMD Millipore Corp.	Cat# 817072

**Critical commercial assays**		

Adenosine Assay Kit (Fluorometric)	Abcam	Cat# ab211094
BD Cytofix/Cytoperm Fixation/Permeabilization Kit	BD Biosciences	RRID: AB_2869008
Duolink® *In Situ* Detection Reagents Green	Sigma-Aldrich	Cat# DUO92014
Duolink® *In Situ* PLA® Probe Anti-Mouse MINUS	Sigma-Aldrich	Cat# DUO92004
Duolink® *In Situ* PLA® Probe Anti-Mouse PLUS	Sigma-Aldrich	Cat# DUO92001
Fast SYBR Master Mix	Thermo Fisher Scientific	Cat# 4385616
FITC Annexin V Apoptosis Detection Kit with 7-AAD	BioLegend	Cat# 640922
Glucose Uptake Glo assay	Promega	Cat# J1341
High Capacity cDNA Reverse Transcription Kit	Thermo Fisher	Cat# 4368814
MILLIPLEX MAP Mouse Cytokine/Chemokine Magnetic Bead Panel - Immunology Multiplex Assay	Merck Millipore	Cat# MCYTOMAG-70K
MojoSort Mouse CD4 Naive T cell isolation Kit	BioLegend	Cat# 480040
Mouse IFN-gamma DuoSet ELISA	R & D systems	Cat# DY485
Mouse IL-1 beta/IL-1F2 DuoSet ELISA	R & D systems	Cat# DY401
NativePAGE Novex 3–12% Bis-Tris Protein Gels	Life Technologies	Cat# BN1001BOX
Non radioactive HPRT Assay Kit	NovoCIB	Cat# K0709-01-2
Novex ECL HRP Chemiluminiscent reagent	Thermo Fisher Scientific	Cat# WP20005
PRPP-Synthetase Superactivity Assay Kit	NovoCIB	Cat# K0709-04-2
Tamoxifen diet	Envigo	Cat# TD.55125

**Experimental models: Organisms/strains**		

Mouse: C57BL/6J	The Jackson Laboratory	RRID: IMSR_JAX:000664
Mouse: IF1- floxed	José M. Cuezva	Esparza-Molto et al.^25^
Mouse: B6.Cg-Tg(Cd4-cre)1Cwi/BfluJ	The Jackson Laboratory	RRID: IMSR_JAX:022071

**Oligonucleotides**		

Please refer to Table S2	N/A	N/A

**Software and algorithms**		

BD FACSDiva 6.6.2	BD Biosciences	RRID:SCR_001456
CFX Maestro Software v2.3	Bio-Rad	RRID:SCR_019145
FlowJo v10	Tree Star	RRID:SCR_008520
GraphPad Prism7	GraphPad	RRID: SCR_002798
ImageJ	NIH	RRID: SCR_003070
*In Vivo* Imaging Software v3.2	Perkin Elmer	RRID:SCR_014247
Omega FLUOstar Control Reader	BMG LABTECH	RRID:SCR_019152
Omega MARS Data Analysis	BMG LABTECH	RRID:SCR_021015
Seahorse Wave v2.4	Agilent technologies	RRID: SCR_014526

**Other**		

Nitrocellulose membrane, Amersham Protran 0.2mm NC	GE Healthcare	Cat# 15289804
PVDF membrane, Immobilon-P, 0.45uM	Merck KGaA	Cat# IPVH00010
XFe96 Flux Pack, Seahorse Bioscience	Agilent Technologies	Cat# 100867-100

### Resource availability

#### Lead contact

Further information and requests for resources and reagents should be directed to and will be fulfilled by the lead contact, José M. Cuezva (jmcuezva@cbm.csic.es).

#### Materials availability

The CD4-Atpif1-KO mouse model developed (Knockout of IF1 in CD4 T lymphocytes) has been safely archived and listed on the INFRAFRONTIER website https://www.infrafrontier.eu/emma/strain-search/straindetails/?q=15299 of the European Mouse Mutant Archive (EMMA) repository.

Other materials generated in this study are available through the lead contact upon request.

#### Data and code availability

• Data reported in our paper is available upon request from the lead contact.
• This paper does not report original code.
• Any additional information required to reanalyze the data reported in this work paper is available from the lead contact upon request.

### Experimental model and study participant details

#### Mice

Mouse experiments were carried out after approval of the institutional review board (Ethical Committee of the UAM, CEI-101-1891-A325; CM PROEX 233/19) in compliance with animal policies and ethical guidelines of the European Community. Mice were housed in the Animal Facility of the CBMSO with a 12-h light/12-h dark cycle and temperatures of 18–23°C with 40–60% humidity.

Conditional IF1 knockout mice (*Atp5if1-*KO) in T lymphocytes (CD4^+^ IF1-KO) were obtained by breeding the IF1-floxed mice^25^ with the B6.Cg-Tg(Cd4-cre)1Cwi/BfluJ (The Jackson Laboratory) mouse line. The latter expresses the Cre recombinase in CD4^+^ and CD8^+^ T lymphocytes. Mice were maintained on C57BL/6J background. Two-Three month-old male and female mice were used except in ageing experiments and otherwise indicated. Wild-type and IF1-floxed alleles were distinguished with 5′-TGCCTGACATTGGTATTGGG-3′ and 5′-GTGCAGCTTGTGGGAGTCAG-3′ primers.^25^ The transgene encoding the Cre recombinase was detected with 5′-CAATTTACTGACCGTACAC-3′ and 5′-TAATCGCCATCTTCC AGCAG-3′ primers. No influence of gender is reported.

#### Ethical considerations

All animal studies were performed following EU ethical and ARRIVE guidelines. Animal procedures have the approval of the Institutional Review Board (UAM University) CEI-101-1891-A325 and Madrid Community PROEX 233/19 Ethical Committees, Spain.

#### CD4^+^ T cells isolation, activation and differentiation

Naive CD4^+^ T cells were obtained from spleen and peripheral lymph nodes of CRL and IF1-KO mice by negative selection using the MojoSort™ Mouse CD4 Naïve T Cell Isolation Kit (BioLegend). For *in vitro* activation, naive CD4^+^ T cells were incubated in the presence of 2 μg/ml plastic-bound purified anti-CD3, 1 μg/mL soluble anti-CD28, and 5 ng/mL recombinant mouse IL-2 during 48 h. For Th1 differentiation, culture medium was also supplemented with IL-12 (10 ng/mL) and anti-IL-4 (4 μg/mL). For Treg cell differentiation, TGF-β (10 ng/mL) was also added to culture medium. For Th17 differentiation, IL-2 was replaced and anti-IL-4 (5 μg/mL), anti-IFN-γ (5 μg/mL), TGF-β (5 ng/mL), IL-23 (20 ng/mL), and IL-6 (20 ng/mL) were incorporated in the culture medium.^43^ Cells were differentiated for 4 days in a humidified incubator at 37°C with a controlled atmosphere of ambient air 10% CO_2_.

### Method details

#### Flow cytometry analysis

For analysis of extracellular markers, spleen, peripheral lymph node, blood or thymus harvested cells were resuspended in PBS staining buffer (PBS supplemented with 1% BSA and 0.02% sodium azide) and the appropriate antibody cocktails at 4°C during 30 min. Then, cells were washed with PBS staining buffer and resuspended in a final volume of 200 μL with 0.1 μg/mL DAPI to analyze the samples by flow cytometry. For intracellular cytokine and nuclear factor staining, harvested cells were stimulated *in vitro* for 4 h with plate-coated anti-CD3, phorbol 12-myristate 13-acetate (50 ng/mL), ionomycin (500 ng/mL) and Brefeldin A (2 μg/mL). Following extracellular staining, cells were washed and resuspended in permeabilization-fixation solution (BD Cytofix/Cytoperm Kit), and intracellular cytokine staining was performed with appropriate fluorescently labeled antibodies following the manufacturer’s protocol. Data were acquired on a FACSCanto II cytometer (Becton Dickinson) and analyzed using the FlowJo software (Tree Star Inc).

For analysis of the different lymphocytic subsets, the following cocktails of antibodies were used: for CD4^+^ cells anti-CD45, anti-CD3 and anti-CD4; for CD8^+^ cells anti-CD45, anti-CD3 and anti-CD8; for Th1 subset anti-CD45, anti-CD4 and anti-IFNγ; for Th2 subset anti-CD45, anti-CD4 and anti-IL-4; for Th17 subset anti-CD45, anti-CD4 and anti-IL-17; for Treg subset anti-CD45, anti-CD4 and anti-FOXP3; for M1 macrophages anti-CD45, anti-CD11b, anti-Ly-6C^high^ and anti-F4/80^low^; for M2 macrophages anti-CD45, anti-CD11b, anti-Ly-6C^low^ and anti-F4/80^high^.

#### Determination of ATP synthase activity

Digitonin-permeabilized activated CRL and IF1-KO CD4^+^ cells were used for the determination of the oligomycin-sensitive mitochondrial production of ATP.^28^^,^^81^ Cells were permeabilized with 50 μg mL^−1^ digitonin in respiration buffer (225 mM sucrose, 10 mM KCl, 5 mM MgCl_2_, 0.05% w/v BSA, 10 mM potassium-phosphate buffer, 1 mM EGTA and 10 mM Tris-HCl; pH 7.4) supplemented with EDTA-free protease and phosphatase inhibitory cocktails and added to a luminometer plate-reader (Omega FLUOstar, BMG LABTECH). ATP production in the absence or presence of 2 μM oligomycin was measured as luminescence production in respiration buffer containing 0.1 mM ADP, 5 mM succinate, 0.15 μM P1,P5-di(adenosine-5′) pentaphosphate, 0.165 mg/mL of luciferin and 0.003 mg/mL luciferase. Relative light units were converted to ATP concentration using an ATP standard curve prepared in respiration buffer.^28^^,^^81^

#### Assessment of ATP hydrolytic activity of ATP synthase in Clear-native (CN) gels

Cells were suspended in 50 mM Tris-HCl pH 7.0 containing 1 M 6-aminohexanoic acid at a final concentration of 10 mg/mL and solubilized with 10% digitonin (4:1 digitonin:protein). 0.1% Ponceau Red and 5.5% glycerol in 1 M 6-aminohexanoic acid was added to the solubilized membranes. Native PAGE™ Novex® 3–12% Bis-Tris Protein Gels (Life Technologies, BN1001BOX) were loaded with 50 μg of protein.^28^ The electrophoresis was performed at a constant voltage of 70 V for 15 min, followed by 1 h at a constant amperage of 10 mA. Cathode buffer: 50 mM Tricine, 15 mM Bis-Tris, 0.05% sodium deoxycholate, pH 7.0, Anode buffer: 50 mM Bis-Tris, pH 7.0. After fractionation, gels were incubated with 270 mM glycine, 35 mM Tris, 8 mM ATP, 14 mM MgSO_4_, 0.2% Pb(NO_3_), pH 8.4 to assess the hydrolytic activity of ATP synthase, which correlates with the formation of white precipitates of lead phosphate as a result of ATP hydrolysis. 2 μM Oligomycin was used to inhibit enzyme activity.

#### Cellular respiration and rates of glycolysis

Oxygen consumption rates (OCRs) were determined in Seahorse XFe96 Extracellular Flux Analyzer (Agilent Technologies). CRL and IF1-KO CD4^+^ cells were seeded in precoated poly-L-lysine plates and equilibrated with Seahorse XF base medium (Agilent Technologies) supplemented with 10 mM glucose, 2 mM glutamine and 1 mM pyruvate for 1 h before assay at 37°C in a CO_2_-free incubator. Mitochondrial function was determined through sequential addition of 6 μM oligomycin, 1 μM 2,4-dinitrophenol (DNP) and 1 μM antimycin A plus 1 μM rotenone following the XF Cell Mito Stress Test injection protocol designed by the manufacturers.

The rates of glycolysis were determined by the enzymatic quantification of lactate concentrations in the culture medium.^17^ In brief, CRL and IF1-KO CD4^+^ cells were activated in activation medium at 4% FBS. Samples of culture medium were taken at 48 h post-activation and precipitated with 4 volumes of cold perchloric acid, incubated on ice for 1 h and then centrifuged for 5 min, 11,000 ×g at 4 °C to obtain a protein-free supernatant. The supernatants were neutralized with 20% (w/v) KOH and centrifuged at 11,000 ×g and 4 °C for 5 min to sediment the KClO_4_ salt. Lactate levels were determined spectrophotometrically (Omega FLUOstar, BMG LABTECH) following the reduction of NAD^+^ at A_340_ after the addition of 4 units of LDH.

#### Cellular glucose uptake

Glucose uptake rates in CRL and IF1-KO activated CD4^+^ cells were determined with the Glucose Uptake-Glo Assay following the manufacturer’s instructions.

#### Determination of ΔΨm and mtROS production

ΔΨm and mtROS production were determined in activated CD4^+^ cells by flow cytometry after staining the cells with 50 nM TMRM or 2.5 μM MitoSOX probes respectively.^29^ DAPI was used to exclude dead cells. The fluorescence intensity of at least 10,000 events was determined in a FACS Canto II cytometer (Becton Dickinson) and analyzed using the FlowJo software (Tree Star). Specificity of TMRM staining was assessed by the addition of 1 μM FCCP once recorded the basal TMRM fluorescence.

#### Cell death and cellular proliferation

Cell death was analyzed at 48 h of cell activation by flow cytometry in CRL and IF1-KO activated CD4^+^ cells with the FITC Annexin V Apoptosis Detection Kit with 7-AAD following manufacturer’s instructions. Cellular proliferation was determined by flow cytometry using Cell Trace Violet. For that, CRL and IF1-KO CD4^+^ cells were isolated, stained with 2 μM Cell Trace Violet and cultured in activation conditions during 48 h. For both assays, the fluorescence intensity of at least 10,000 events was determined in a FACS Canto II cytometer (Becton Dickinson) and analyzed using the FlowJo software (Tree Star).

#### Determination of adenosine

The adenosine released into the culture medium of CRL and IF1-KO CD4^+^ cells at 48 h of activation was determined using the fluorometric Adenosine Assay Kit (Abcam), following the manufacturer’s instructions. Fluorescence was measured in 96-well plates with FLUOstar Omega (BMG Labtech).

#### Determination of phosphoribosyl pyrophosphate (PRPP) synthetase and hypoxanthine-guanine phosphoribosyl transferase (HPRT) enzymatic activities

48 h-activated CRL and IF1-KO CD4^+^ cells were homogenized in 8 volumes of 10 mM KH_2_PO_4_ pH 7 buffer using a glass-teflon potter. After 30 min incubation at 4 °C, the homogenates were centrifuged at 16,000 × g for 30 min at 4 °C. PRPP-synthetase activity was determined in the supernatant with the PRECICE® PRPP-S Assay Kit. HPRT activity was determined with the PRECICE® HPRT Assay Kit. A_340_ was measured in 96-well plates with FLUOstar Omega (BMG Labtech).

#### Determination of mtDNA copy number

Total genomic DNA (nuclear and mitochondrial) was extracted from CRL and IF1-KO CD4^+^ cells with phenol:chloforofom:isoamyl alcohol (25:24:1) method. Mitochondrial/nuclear DNA ratio was quantified with Fast SYBR Master Mix in a CFX Opus 384 Real-Time PCR System (Bio-Rad) at the Genomics and NGS Core Facility (CBMSO). Thermal cycling conditions were as follows: initial denaturation of 20 s at 95°C, 40 amplification cycles of 1 s at 95°C, and 20 s at 60°C each, followed by a dissociation curve analysis to detect possible nonspecific amplification. Standard curves with serial dilutions of pooled DNA were used to assess amplification efficiency of the primers and to establish the dynamic range of DNA concentration for amplification, which was 10 ng per run. The relative copy number of mtDNA molecules was determined with the comparative ΔΔCT method using the mean of the single copy nuclear genes nB2M, nAtp5b, nActb and nSdha and the mean of the single copy mitochondrial genes mt-12S, mt-16S, mt-Cytb and mt-Co2. The sequence of the primers used is displayed in Table S2.

#### RT-PCR analysis

RNA was extracted and purified from CRL and IF1-KO CD4^+^ cells with Trizol reagent (Invitrogen) according to the manufacturer’s instructions. Reverse transcription reactions were performed using 500 ng of total RNA and the High Capacity cDNA Reverse Transcription Kit. Real-time PCR was done with Fast SYBR Master Mix in a CFX Opus 384 Real-Time PCR System (Bio-Rad) from the Genomics and NGS Core Facility (CBMSO). Thermal cycling conditions were as follows: initial denaturation of 20 s at 95°C, 40 amplification cycles of 1 s at 95°C, and 20 s at 60°C each, followed by a dissociation curve analysis. Standard curves with serial dilutions of pooled cDNA were used to assess amplification efficiency of the primers and to establish the dynamic range of cDNA concentration for amplification, which was 8 ng of input RNA per run. The relative expression of the mRNAs was determined with the comparative ΔΔCT method using *18S* and *β-actin* as housekeeping genes. Primers used to amplify the target genes are listed in Table S2.

#### Protein extraction and Western blot analysis

Cells or mouse tissues were lysed in Tissue Protein Extraction Reagent (T-PER, Thermo Fisher) supplemented with EDTA-free protease and phosphatase inhibitory cocktails. Homogenates were freeze-thawed three times in liquid nitrogen and clarified by centrifugation at 11,000 ×g for 30 min at 4°C. Protein concentration was determined using Bradford reagent (Bio-Rad protein assay, Bio-Rad). The resulting protein extracts were fractionated on SDS-12% PAGE and transferred onto PVDF or nitrocellulose membranes for immunoblot analysis. Membranes were blocked with 5% nonfat dried milk in TBS with 1% Tween 20 for 1 h and incubated with the primary antibodies diluted in 3% BSA in TBS overnight at 4°C. The primary antibodies used are rabbit anti-IF1,^46^ rabbit anti-β-F1,^40^ mouse anti-α-tubulin, mouse anti-CD4 and rabbit anti-APRT. Peroxidase-conjugated anti-mouse or anti-rabbit IgGs (1/5,000) were diluted in TBS with 1% Tween 20 and used as secondary antibodies. The Novex® ECL system (Thermo Fisher) was used to visualize the bands. The intensity of the bands was quantified using a GS-900™ Calibrated Densitometer (Bio-Rad) and the Analyze Gel command of ImageJ software (NIH).

#### Determination of ATP synthase oligomers by proximity ligation assays (PLA)

The method followed was recently described,^28^ taking into consideration that ATP synthase only has one γ-F1-ATPase subunit per monomer of enzyme. When it is assembled into oligomers, the molecular distances between γ-F1-ATPase subunits fall below the molecular distances required for positive amplifications of PLA.^28^ CRL and IF1-KO CD4^+^ cells were seeded on pre-coated poly-L-lysine coverslips, fixed with 4% paraformaldehyde (PFA) and then permeabilized with 0.1% Triton X-100. Next, Duolink® PLA Probes and Fluorescent Detection Reagents (Sigma-Aldrich) were used following manufacturer’s protocol. In brief, cells were blocked with Duolink Blocking Solution and incubated with the primary anti-γ-F1-ATPase antibody as unique primary antibody (1/250). After overnight incubation, PLA anti-mouse PLUS and MINUS probes were added for 1 h at 37°C. For amplification, the samples were processed following manufacturer’s instructions. Samples were mounted *in situ* with DAPI-containing mounting medium. Cellular fluorescence was analyzed by confocal microscopy in an A1R+ microscope (Nikon) at CBMSO Optical and Confocal Microscopy Facility and processed with ImageJ software. For the analysis of the PLA signals per cell, the maximum intensity of the z-stacks comprising the cells was projected and the number of dots and nuclei were counted with the analyze particles command in thresholded images.

#### ELISA analysis of cytokines

Blood concentration of IL-1β and IFNγ cytokines in CRL and CD4-IF-KO LPS-injected mice was determined by Enzyme-Linked ImmunoSorbent Assays (ELISA) using Mouse IL-1 beta/IL-1F2 DuoSet ELISA and Mouse IFN-gamma DuoSet ELISA kits, respectively, following manufacturer’s instructions.

#### Multiplexed quantitative analysis of cytokines

Mouse tissues were lysed in T-PER supplemented with EDTA-free protease and phosphatase inhibitory cocktails using the Bead Mill 24 Homogenizer system (Fisherbrand). Homogenates were clarified by centrifugation at 11,000×g for 30 min at 4 °C. Protein concentration was determined with Bradford reagent (Bio-Rad Protein Assay). The levels of cytokines in the serum and tissues from mice were analyzed following the protocol of Mouse T Cell Kit, MILLIPLEX MAP Assay.

#### Electron microscopy

Sample preparation was carried out by the CBMSO Electron Microscopy Facility. Briefly, the CD4^+^ cellular pellet was fixed at room temperature with 4% PFA-2% glutaraldehyde (GLA) in 0.1 M phosphate buffer pH 7.4 for 2 h. Postfixation was carried out with 1% OsO_4_– 0.8% K_3_Fe(CN)_6_–water at 4°C for 1 h and then the samples were embedded in gelatin matrix (10% in bidistilled water) and kept on ice until gelatin solidified. Then, samples were cut in 1 mm^3^ cubes, dehydrated with ethanol and embedded in epoxy TAAB 812 resin (TAAB Laboratories). After resin polymerized, 70-nm-thick (ultrathin) sections were obtained and stained with uranyl acetate and lead citrate. Images were examined at 80Kv in a Jeol JEM1400 Flash Transmission Electron Microscope and a CMOS Oneview (4Kx4K) camera (Gatan). Mitochondrial shape descriptors were calculated manually in ImageJ (NIH). Each individual mitochondrion was surrounded using the freehand selection tool by following the outer membrane; the Area and Circularity were measured using the corresponding plugins under Measure command.

#### Mouse treatments

##### DSS-induced colon inflammation

Four-month-old CRL and CD4-IF-KO male mice were treated with 2.5% DSS (PanReac AppliChem) added in the drinking water to induce acute intestinal inflammation.^27^ The weight of mice was recorded every day as a measure of animal welfare.

##### Lipopolysaccharide (LPS)-induced inflammation

LPS from *Escherichia coli* (6 mg/kg of mice) was intraperitoneally injected to three-month-old CRL and CD4-IF-KO male mice. 6 h after injection, blood was drawn by mandibular puncture to analyze cytokine levels. Weight determination was daily determined until the day of sacrifice as a measure of animal welfare.

##### Tamoxifen administration

CRL mice were treated with tamoxifen in the chow in two cycles of five *days* per week before sacrifice.

##### BSA/CFA immunization

Three-month-old CRL mice were injected with 200 μL of 100 μg/mL BSA emulsified with complete Freud adjuvant (1:1) to obtain an inflammatory response, or with PBS as control. 100 μL were injected intraperitoneally and 50 μL were injected subcutaneous in each groin. Mice were sacrificed 10 days after the injections.

#### Hematology analysis

Blood from CRL and CD4-IF-KO mice was collected in EDTA tubes and analyzed using an Element HT5 Hematology Analyzer (Heska).

#### Glucose tolerance test

Four-month-old CRL and CD4-IF-KO male mice were injected intraperitoneally with a glucose solution (0.2 g/mg of mouse weight) after 12 h of starvation. The blood glucose concentration was measured using the One Touch Select Plus strips (Johnson & Johnson) before and after injection at the indicated time intervals.

### Quantification and statistical analysis

The results shown are the mean ± SEM. All tests were performed in a non-blinded fashion. Statistical analyses were performed by Student’s t-test or one-way ANOVA with a post hoc Tukey’s test. Survival curves were derived from Kaplan–Meier estimates and compared by log-rank test. Statistical analyses were calculated using GraphPad Prism v8. Values of p< 0.05 were considered statistically significant. Specific statistical details and methods used in each experiment can be found in figure legends. P values are provided in figure legends (∗p< 0.05, ∗∗p< 0.01, ∗∗∗p< 0.001). The n used in each statistical test is indicated in the figure legends, and when not specified, n refers to the animals or sample size per genotype.

## Supplemental information

Document S1. Figures S1, S2 and Table S1

Table S2. Primers used for the determination of mtDNA content and RT-PCR analysis Primers were self-designed with the online interface Primer3Plus and acquired to Integrated DNA Technologies (IDT)

Data S1. Original data files, related to Figure 1

Data S2. Original data files, related to Figure 2

Data S3. Original data files, related to Figure 3

Data S4. Original data files, related to Figure 4

Data S5. Original data files, related to Figure S1

Data S6. Original data files, related to Figure S2

Data S7. Original data files, related to Table S1
