# Supplementary material for: Ablation of Atp5if1 impairs metabolic reprogramming and proliferation of T lymphocytes and compromises mouse survival
Source: iScience. 2024 May 3;27(6):109863. doi: 10.1016/j.isci.2024.109863 (PMC11126974; doi:10.1016/j.isci.2024.109863)
Supplement: Document S1. Figures S1, S2 and Table S1 [file mmc1.pdf]

iScience, Volume *27*

Supplemental information

Ablation of *Atp5if1* impairs metabolic reprogramming and proliferation of T lymphocytes and compromises mouse survival

Inés Romero-Carramiñana, Sonia Dominguez-Zorita, Pau B. Esparza-Moltó, and José M. Cuezva

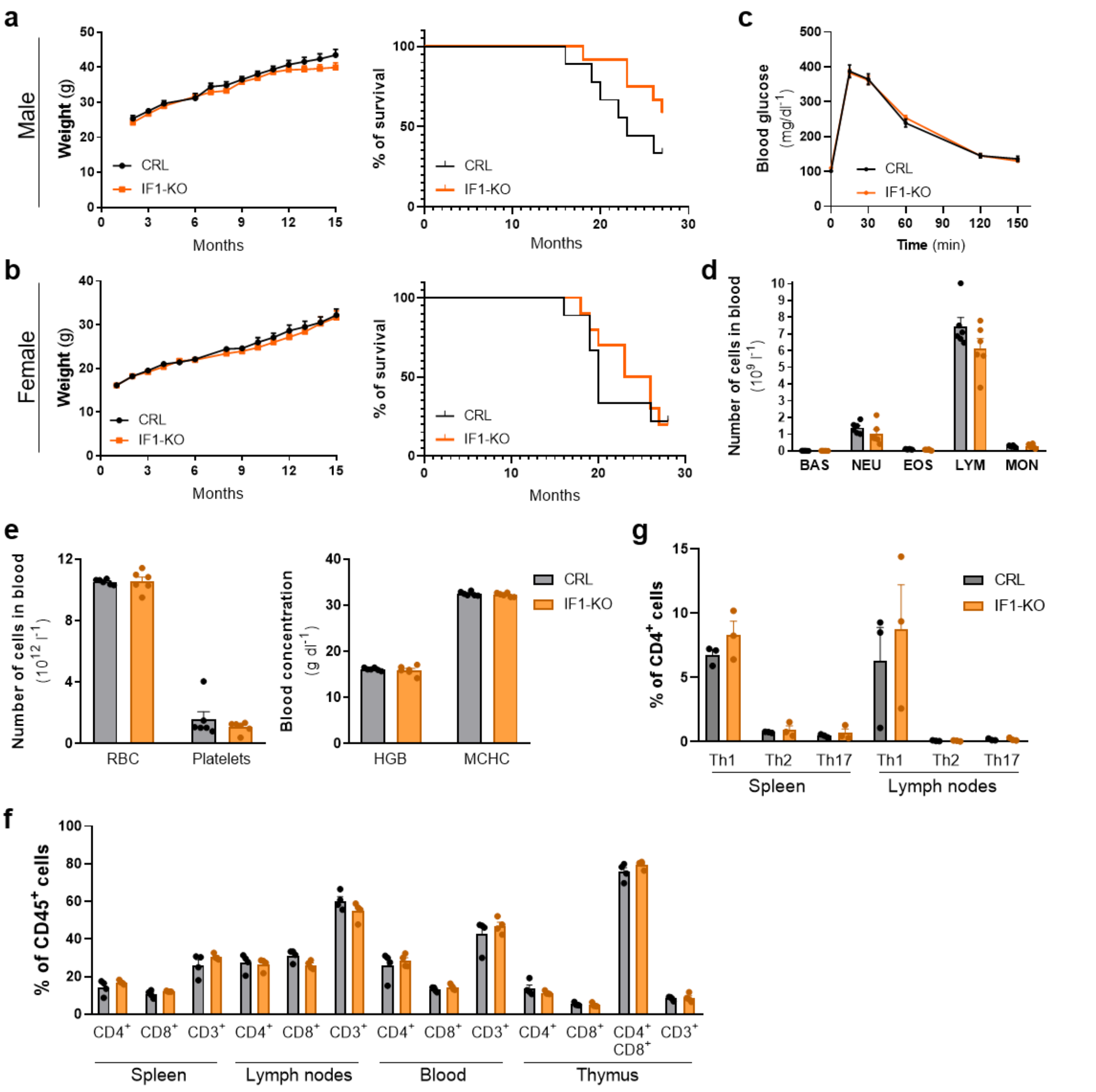

Supplemental Figure S1. CD4$^+$ IF1-KO mice show no alterations in longevity, metabolic and hematologic parameters under non-stressed conditions, related to Figure 1. a-b, Left, plots showing the weight of CRL and IF1-KO male (**a**) and female (**b**) mice. Right, shows the corresponding Kaplan–Meier survival analysis of male (**a**) and female (**b**) mice (n = 9-12). **c,** Glucose Tolerance Test (GTT) of CRL and IF1-KO male mice (n = 6). **d-e,** Hematologic analysis of CRL (n=6) and IF1-KO male mice (n= 6), using an Element HT5 Hematology Analyzer. **d**, Number of basophils (BAS), neutrophils (NEU), eosinophils (EOS), lymphocytes (LYM) and monocytes (MON) in blood (n = 6). **e,** Left, number of red blood cells (RBC) and platelets in blood. Right, hemoglobin concentration (HGB) and hemoglobin per RBC (MCHC). **f,** Percentage of CD4$^+$ (CD4$^+$;CD45$^+$;DAPI$^-$), CD8$^+$ (CD8$^+$;CD45$^+$;DAPI$^-$) and CD3$^+$ (CD3$^+$;CD45$^+$;DAPI$^-$) cells in spleen, lymph nodes, blood and thymus of CRL and IF1-KO mice (n= 4). **g,** Percentage of Th1 (INFγ^+;CD4$^+$;CD45$^+$), Th2 (IL4$^+$;CD4$^+$;CD45$^+$) and Th17 (IL17$^+$;CD4$^+$;CD45$^+$) cells in spleen and lymph nodes of CRL and IF1-KO mice (n = 3). The histograms show the mean and the error bars ± SEM.

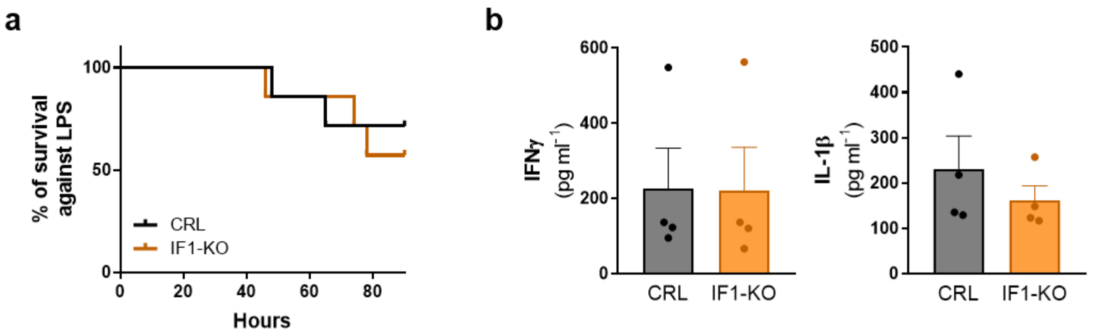

Supplemental Figure S2. CD4$^+$ IF1-KO mice withstand an LPS-induced endotoxemia, related to Figure 4. a, Kaplan–Meier survival analysis of CRL (n=7) and IF1-KO mice (n = 7) after lipopolysaccharide (LPS)-induced inflammation. **b,** Histograms show the mean ± SEM of IFNγ and IL-1β levels in serum of CRL (n=4) and IF1-KO mice (n = 4) six hours after lipopolysaccharide (LPS)-induced inflammation.

Supplemental Table S1, related to Figure 4. Cytokine concentrations in serum, spleen and colon of CRL and IF1-KO mice at day 6 of DSS-treatment. Data are expressed as mean ± SEM. N.a., below the detection limit. *$p \leq 0.05$ when compared to CRL by Student's t test.

	Serum		Spleen		Colon	
	**CRL**	**IF1-KO**	**CRL**	**IF1-KO**	**CRL**	**IF1-KO**
	pg / ml		pg / mg of protein		pg / mg of protein	
**INFγ**	9.20 ± 2.7	19.33 ± 4.1*				
**IL-1α**	697.97 ± 45.9	782.96 ± 83.1				
**IL-1β**	16.82 ± 4.8	13.32 ± 5.6	0.31 ± 0.03	0.37 ± 0.07	3.63 ± 1.53	2.21 ± 0.72
**TNFα**	6.46 ± 1.2	5.19 ± 0.3	n.a	n.a	0.81 ± 0.32	0.33 ± 0.04
**IL6**	162.04 ± 64.2	151.55 ± 73.1				
**IL2**	15.48 ± 1.7	9.53 ± 3.1	0.22 ± 0.03	0.15 ± 0.02	0.22 ± 0.02	0.16 ± 0.02
**IL10**	9.28 ± 1.9	6.94 ± 2.3				
**IL17**	4.53 ± 0.7	4.82 ± 0.9	n.a	n.a	0.84 ± 0.44	0.36 ± 0.11
**GM-CSF**	25.99 ± 7.6	20.57 ± 8.5	n.a	n.a	0.19 ± 0.08	0.14 ± 0.04
**M-CSF**	18.48 ± 6.2	15.33 ± 5.4				
**MCP1**	45.48 ± 6.0	39.40 ± 8.7	0.72 ± 0.26	0.74 ± 0.21	40.97 ± 17.60	13.30 ± 7.08
**IL12**	12.03 ± 2.8	10.88 ± 3.4	n.a	n.a	n.a	n.a
**IL5**	10.49 ± 2.4	10.76 ± 2.7	n.a	n.a	n.a	n.a
**IL13**	106.36 ± 5.3	101.66 ± 15.0	n.a	n.a	n.a	n.a
