# Supplementary material for: Ablation of Atp5if1 impairs metabolic reprogramming and proliferation of T lymphocytes and compromises mouse survival
Source: iScience. 2024 May 3;27(6):109863. doi: 10.1016/j.isci.2024.109863 (PMC11126974; doi:10.1016/j.isci.2024.109863)
Supplement: Table S2. Primers used for the determination of mtDNA content and RT-PCR analysis — Primers were self-designed with the online interface Primer3Plus and acquired to Integrated DNA Technologies (IDT) [file mmc2.pdf]

Supplemental Table S2. Primers used for the determination of mtDNA content and RT-PCR analysis. Primers were self-designed with the online interface Primer3Plus and acquired to Integrated DNA Technologies (IDT).

	Forward (5'-3')	Reverse (5'-3')
nB2M	CCGGATTGGCTGTGAGTT	GACAAGCACCAGAAAGACCAG
nAtp5b	CCTGCTGATGACCTGACTGA	GCTGGATAGATGCCCAACTC
nActb	ACCCAGAGAGCTCACCATTC	TGATCCACATCTGCTGGAAG
nSdha	TACTACAGCCCCAAGTCT	CCGTGAAGACCTCAGCAACT
mt-12S	AAACAGCTTTTAACCATTGTAGGC	TTGAGCTTGAACGCTTTCTTTA
mt-16S	CACTGCCTGCCCAGTGA	ATACCGCGGCCGTTAAA
mt-Cytb	AGTAGACAAAGCCACCTTGA	CCGCGATAATAAATGGTAAG
mt-Co2	CTACAAGACGCCACAT	GAGGGGGAGAGCAAT
If1	CGGACTCGTCGGATAGCA	CTCCTTCGAATGGTGGTCAA
Inf- γ	CACACTGCATCTTGGCTTTG	TCTGGCTCTGCAGGATTTTC
Il17a	CAGACTACCTCAACCGTTCCA	CATGTGGTGGTCCAGCTT T
Tgf- β	ATACGCCTGAGTGGCTGTCT	GGTTCATGTCATGGA TGGTG
Mt-ND1	GCCAGGAAATTGCGTAAGAC	TAGAATGGGGACGAGGAGTG
Mt-ND5	TGATGGTACGGACGAACAGA	CTCCGATGCGGTTATAGAGG
Ndufs3	ATCCTGACAGATTATGGCTTCG	CACTACCCGCTTTACCTCATC
Ndufs7	GGCTACTACCACTACTCCTACT	CTTCTGTTCACGCTTGATCTTC
Ndufa9	GCACGAGACAAAGATTCTATCAG	TCACAAAAACATCCTCAAAATCAAAG
Ndufa10	TCCTGGAGGCAATGTACAAC	TCGATATAGATGACTGCGTGTG
Sdha	TCCATACACCGAATAAGAGCAAA	ACCAGCCCTAGTGACCAT
Sdhb	CTGTACGAGTGCATCCTGTG	TCCTCTGTGAAGTCGTCTCT
Etfdh	CGCCTCTCTCCTTTGTATCTG	CTTGGAGCACACAGAGGTAG
Uqcrc1	GCCACTATGACTGCACTTACG	GCCCAGTATCAGAGTAGGAGA
Uqcrc2	GCTAATCCTTTGTACTGTCCTGA	AGACAGAATGACTCACACCAAG
Cox4i1	GAGAGCCATTTCTACTTCGGT	GCAGACAGCATCGTGACAT
Cox5a	CATCCAGGAACTTAGACCAACT	TTTCCAGGCAACTGTTTCAATC
Cox8a	CATCTTGACTCCCTGACCTTG	CTTCGAGTGGACCTGAGC
Atp5f1a	TTCAAGCAGAGGAGATGGTG	CATTGTCGGGTTCCAAGTTC

Atp5f1c	CCGAGTGTATGGGACAGGTT	GCACCACAAAGCCCTCTATC
Atp5mc1	GGCACAGTGTTTGGTAGCTTG	CCCAGAATGGCATAGGAGAA
Atp5pd	ATGCCCTGAAGATTCCTGTG	TCCAGCTGCTTCTCATACTCC
β-f1	TCCACTGGACTCCACCTCTC	CCGTGAAGACCTCAGCAACT
Opa1	AGCATTTCGAGCAACAGATCA	CGCTCCAAGATCCTCTGATAC
Dnm1l	GCAACATCAGAAGCACTCAAG	AACCCTTCCCATCAATACATCC
Mic10	GAAGCTAGGTACTGGGTTTGGA	GCCAGAACCAAAGGCTAATG
Mic60	GTGGCACAGAGCCAGAAAA	CCGGGACCGAAAGTGTATC
Oma1	GCAAAGAACACTTCCGACTTC	TTCTTTCACAGTCAGGTAGCG
Yme1l1	GTATTCAAGGCAGACGATCAATC	GACCAGGACGTATTAAGGCATT
Timm22	AAGAACAGCGTCATCAGTGG	CAGCAGAGAAAGCAGCAA
Timm23	GATGACCTCAACACAGTAGCAG	GTGCATAGAGACTGGTGAGTG
Tomm20	TCCTTGAAGAGATACAGCTTGG	CAGCAATGGCATTTGTCAGG
Tomm40	GAGCAACCGTTTCCAGGT	CACTATTGTCCATGTCACCCA
Hsp60	TCTTCAGGTTGTGGCAGTCA	CCCCTCTTCTCCAAACACTG
18S	AACGGTCTAGACAACAAGCTG	AGTGGTCTTGGTGTGCTGAC
β-actin	AACACAGTGCTGTCTGGTGGT	GATCCACATCTGCTGGAAGG
